# Extracellular Vesicles Released During Normothermic Machine Perfusion Are Associated With Human Donor Kidney Characteristics

**DOI:** 10.1097/TP.0000000000004215

**Published:** 2022-11-22

**Authors:** Wouter W. Woud, Asel S. Arykbaeva, Ian P.J. Alwayn, Carla C. Baan, Robert C. Minnee, Martin J. Hoogduijn, Karin Boer

**Affiliations:** 1 Department of Internal Medicine, University Medical Center Rotterdam, Erasmus MC Transplant Institute, Rotterdam, The Netherlands.; 2 Department of Surgery, Leiden University Medical Center, Leiden, The Netherlands.; 3 Transplant Center, Leiden University Medical Center, Leiden, The Netherlands.; 4 Division of Hepato-Pancreato-Biliary and Transplant Surgery, Department of Surgery, Erasmus MC Transplant Institute, Erasmus MC, University Medical Center Rotterdam, Rotterdam,The Netherlands

## Abstract

Extracellular vesicles (EVs) are tissue-specific particles released by cells containing valuable diagnostic information in the form of various biomolecules. The characterization of EVs released by kidney grafts during normothermic machine perfusion (NMP) may present a promising avenue to assess graft status before transplantation.

**Methods.:**

We phenotyped and determined the concentrations of EVs in the perfusate of 8 discarded expanded-criteria donor human kidneys during 6 h of NMP. Perfusate samples were taken at 0/60/180/360 min and examined with nanoparticle tracking analysis and imaging flow cytometry (IFCM). Using IFCM, EVs were identified by their expression of common EV markers CD9, CD63, and CD81 (tetraspanins) in combination with either platelet endothelial cell adhesion molecule (CD31), pan-leukocyte protein (CD45), or carboxyfluorescein succiminidyl ester (CFSE) fluorescence.

**Results.:**

Nanoparticle tracking analysis measurements revealed the release of nanoparticles <400 nm into the perfusate during NMP. With IFCM, tetraspanin protein signatures of the released nanoparticles were characterized, and the majority (~75%) of CFSE+ EVs were found to be CD81+, whereas ~16% were CD9+ and ~8% CD63+. Correlation analysis of concentrations of identified EV subsets with crude donor characteristics and NMP viability characteristics revealed significant correlations with cold ischemia time, donor age, and renal flow.

**Conclusions.:**

Our findings demonstrate that discarded expanded-criteria donor kidney grafts release distinct EV subsets during NMP. Because these subsets correlate with well-established indicators of transplant outcome, EVs might represent new potential candidates for assessment of kidney graft quality.

## Graphical abstract

**Figure:**
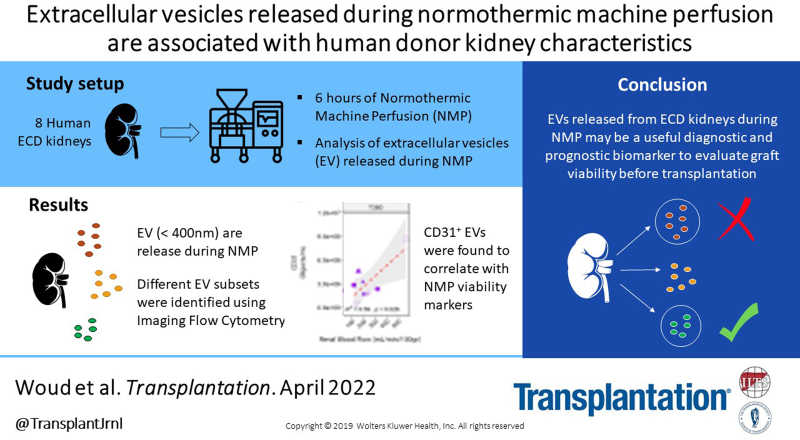

## INTRODUCTION

The shortage of available grafts, the increasing number of patients on the waiting list, and the general aging of the population have led to an increased use of expanded-criteria donor (ECD) grafts as well as grafts procured from donation after circulatory death (DCD).^1^ Both ECD and DCD grafts are associated with poorer transplant outcomes when compared to organs from standard criteria donors.^2,3^ This is in part because older grafts are more susceptible to ischemia-reperfusion injury and because of the inability to fully recover after transplantation as a consequence of natural loss of nephron mass.^4^ Moreover, an essential problem with the usage of these kidneys is the lack of quality measures needed to guide the clinician in deciding whether to accept or decline the organ. Combined, this has forced the transplant community to (1) investigate new methods of organ preservation aimed at reducing ischemia-reperfusion injury and (2) to develop tools to evaluate transplant kidney quality.

The most recent development in organ preservation is normothermic machine perfusion (NMP). In contrast to hypothermic machine perfusion (HMP), NMP aims to restore cellular metabolism and function to the organ, which is achieved through the circulation of a warm, oxygenated red blood cell-based solution through the organ before transplantation.^5,6^ Because metabolism is activated, NMP offers the possibility to assess graft status before transplantation through monitoring of the perfusion dynamics and analysis of biomarkers in the perfusion fluids.^2,5,7,8^

Potential candidates for the assessment of graft status are extracellular vesicles (EVs). EVs are lipid bilayer membrane structures (30–8000 nm in diameter^9^) involved in cellular communication.^10^ They express surface markers and carry a “cargo” (eg DNA/RNA/lipids/proteins^11^), both of which are thought to be indicative for the status of its cell of origin. EVs are excreted by virtually all cell types and are considered an excellent, stable biomarker platform as their cargo is protected from fragmentation and degradation by the lipid bilayer.^12^ In transplantation, levels of (human) donor-specific EVs in animal models have been shown to be associated with acute rejection of the allograft.^13,14^ Additionally, micro RNA, RNA, and proteomic profiling of EVs obtained from kidney preservation fluids^15^ or the urine of kidney recipients^16,17^ suggest that EV analysis might enable kidney health assessment and prognostication in kidney transplantation.

Despite the interest in EVs as a biomarker, the analysis of EVs is hampered by their physical characteristics, such as their small size, low epitope copy number,^18^ the variety of protein markers depending on the cell source, and the confinement of some markers on the luminal side of the vesicles.^19,20^ In the absence of a specific marker, EVs are identified by their expression of common markers, such as CD9, CD63, and CD81. These proteins have a broad tissue distribution, belong to the tetraspanin superfamily, and are enriched in EVs.^21^

Previously, our group was able to quantify the release of nanoparticles (such as protein aggregates and EVs) by ECD kidneys during NMP.^22^ Here we apply our recently developed imaging flow cytometry (IFCM)–based methodology^23^ to identify, phenotype and determine the concentration of EVs ≤400 nm in diameter released by discarded human kidney grafts during NMP. We show the identification of distinct EV subsets based on their tetraspanin profile in combination with the detection of esterase activity, platelet endothelial cell adhesion molecule (CD31), or the pan-leukocyte protein (CD45). Additionally, in the absence of kidney function, we perform a correlation analysis of the identified EV subsets with crude donor and NMP viability characteristics to explore the potential clinical implications of the identified EVs.

## MATERIALS AND METHODS

### Ethical Approval

Ethical approval (number B19.019) for experiments with discarded donor kidneys was granted by the Medical Ethical Committee of the Leiden University Medical Center and University Medical Center Groningen. Research consent was obtained from the relatives of all donors before organ retrieval.

### Procurement, Preparation of the Kidney, and Sample Drawing

**FIGURE 1.**
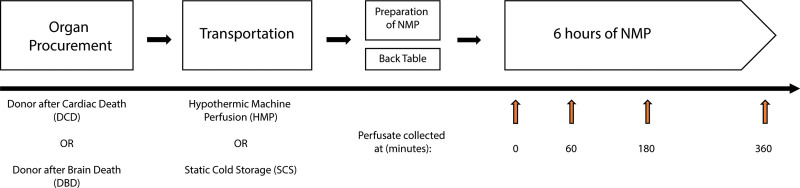
Schematic overview representing procurement, transportation, preparation of the kidneys, and sample drawing. NMP, normothermic machine perfusion.

The included kidney grafts (N = 8) were procured from deceased donors according to the Dutch national guidelines and were deemed untransplantable because of procurement-related factors or factors determined before retrieval (specified in Table 1). After in situ flushing of the abdominal organs with cold University of Wisconsin preservation solution, the kidneys were retrieved, preserved by either static cold storage or HMP, and transported to the participating centers (Leiden University Medical Center or University Medical Center Groningen) where NMP was initiated and performed up to 6 h (Figure 1). Upon arrival at the participating centers, kidney grafts were inspected and prepared for connection to the NMP circuit under sterile conditions, whereas the perfusion machine (Kidney Assist, Organ Assist, Groningen, The Netherlands) was primed. The NMP setup was primed as previously described in the PROPER study.^24^ Kidneys were subjected to subsequent NMP when the cold ischemia time (CIT) did not exceed 24 h at arrival.

**TABLE 1.** Donor and retrieval data

Kidney ID	K1*^a^*	K2*^a^*	K3	K4	K5	K6	K7*^b^*	K8*^b^*
Donor age (y)	71	71	65	53	70	63	75	75
Donor gender (F/M)	M	M	M	M	M	M	M	M
Donor type (DBD/DCD)	DCD	DCD	DBD	DCD	DBD	DCD	DCD	DCD
Cause of death	Circ/CA	Circ/CA	CVA	CVA/CI	Trauma: capitis	Circ/CA	CVA/ICB	CVA/ICB
Warm ischemia time (min)	11	11	–	27	–	19	14	14
Cold ischemia time (h)	2.2	6.4	17.8	14.3	14.3	16.1	11.4	20.2
Initial cold preservation (SCS/HMP)	SCS	SCS	HMP	SCS	SCS	HMP	SCS	SCS
Left/right kidney	Left	Right	Left	Right	Left	Right	Right	Left
Reason for discard	Severe kidney failure	Severe kidney failure	Suspected malignancy	Duodenum perforation	Hepatitis B	Surgical, ureter too short	Medical reasons	Medical reasons
Kidney weight (g)	266	352	377	519	298	308	410	213

*^a-b^*Represent paired kidney grafts from same donor.

CI, cerebral ischemia; Circ/CA, circulational: cardiac arrest; CVA, cerebral vascular bleeding; DBD, donation after brain death; DCD, donation after circulatory death; F, female; HMP, hypothermic machine perfusion; ICB, intra cerebral bleeding; M, male; SCS, static cold storage.

Perfusate samples were drawn before (0) and after 60/180/360 min of NMP, centrifuged at 3700 rpm at room temperature, and the supernatant was stored at −80 °C.

### Nanoparticle Tracking Analysis

Size distribution and concentration of nanoparticles within the perfusates were measured with the Malvern Panalytical NanoSight NS300 and analyzed with nanoparticle tracking analysis (NTA) software version NTA 3.4 Build 3.4.003. Samples were diluted in 0.2 µm filtered phosphate buffered saline until 20–60 particles were in the field of focus during acquisition and 10 videos of 15 s were recorded with camera level 11 and analyzed with detection threshold 5.

### Sample Labelling and Controls

Perfusates were stained with monoclonal antibodies (mAbs) and carboxyfluorescein diacetate succinimidyl ester (CFDA-SE) as extensively described in our previous work^23^ and detailed in **SDC**, http://links.lww.com/TP/C460, Materials and Methods; Sample Labelling.

To ascertain EV measurements the following controls were applied, as recommended by the MIFlowCyt-EV framework^25^: buffer only, buffer with reagents, unstained samples, isotype controls, and detergent treatment, which aims to disrupt the membranous structure of EVs thereby allowing discrimination between biological and artificial events. Detergent treatment was performed by adding 20 µL of a 10% (V/V) TritonX-100 detergent to the samples followed by 30 min of incubation at room temperature before acquisition.

### Data Acquisition and Analysis

All samples were acquired on an ImageStreamX MkII instrument (IS^x^; Luminex). Settings as extensively described in our previous work^23^ and detailed in **SDC**, http://links.lww.com/TP/C460, Materials and Methods; Acquisition were used.

Data analysis was performed using Amnis IDEAS software (version 6.2). To ensure the analysis of EVs we (1) selected all particles with side scatter intensities ≤900 a.u., and (2) identified and excluded coincidence detection by counting the number of fluorescent spots within the pixel grid for each event acquired; events showing multiple spots were excluded from the analysis.^23^ This gating strategy ensures the selection and analysis of single spot fluorescent particles ≤400 nm. Gating areas and cutoffs were established through the identification of (fluorescent) populations in unstained and single stained samples, and arbitrary fluorescent intensities were converted into equivalent molecules of fluorescence (ERF) values based on previously published calibration data.^23^ Lower and upper gating area cutoffs were defined as 677–112,201 ERF for BV421; 35.40–3776 ERF for carboxyfluorescein succiminidyl ester (CFSE); 206–14,770 ERF for phycoerythrin; and 6.40–123 ERF for allophycocyanin.

### Statistical Analysis

Statistical analysis was performed using R version 4.0.2 and RStudio (RStudio Team [2016] RStudio: Integrated Development for R. RStudio, Inc, Boston, MA; URL http://www.rstudio.com/) version 1.1.463. Statistical significance between EV concentrations and binary data was determined through two-sided *t*-tests and 95% confidence intervals with unpaired data. Linear correlations with continuous variables were examined using the Pearson correlation method. R^2^ values ≥0.6 and *P* values <0.05 were considered significant.

## RESULTS

### Kidneys Release Nanoparticles During NMP

**FIGURE 2.**
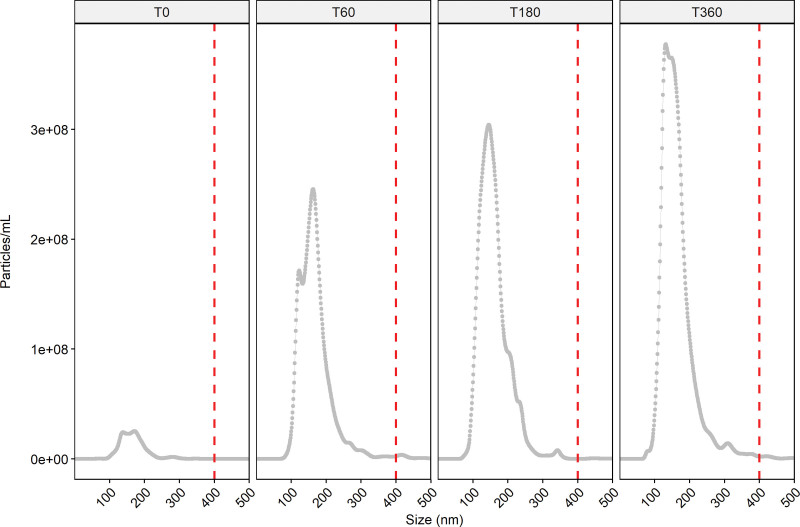
Concentration and size distribution of particles released by a discarded kidney during (60, 180, 360 min—T60/ T180/ T360, respectively) normothermic machine perfusion (NMP), as measured with nanoparticle tracking analysis (NTA). T0 represents particle concentration and size distribution in baseline perfusate. A clear increase in particle concentration was observed during NMP and the majority of released particles were observed to be <400 nm (red striped lines).

To study whether discarded kidneys release nanoparticles during NMP, perfusate samples drawn at 0/60/180/360 min were measured with NTA to determine the particle concentration and size distribution (Figure 2). We observed a baseline concentration of 2.05E^9^ ± 2.13E^8^ particles/mL (mean ± SD and area under the curve) within the perfusate before contact with the kidney (T0, baseline perfusate). Total particle concentrations were observed to increase over time during NMP: 1.96E^10^ ± 7.21E^8^/ 2.54E^10^ ± 7.85E^8^/ 3.06E^10^ ± 6.27E^8^ objects/mL at 60, 180, and 360 min, respectively. Average particle size was established to be <400 nm irrespective of the time of sampling.

### Detergent Treatment Confirms the Analysis of EVs

Next, we stained the perfusate samples of 8 NMP kidneys with CFDA-SE and an anti-tetraspanin antibody mixture (anti-CD9/anti-CD63/anti-CD81) labeled with allophycocyanin and measured the samples with IFCM. CFDA-SE is converted to CFSE by intravesicular esterases and was used to discriminate EVs from contaminating agents such as lipoproteins. Identification and validation of single EV measurement by IFCM are presented in **Figure S1, SDC**, http://links.lww.com/TP/C460.

**FIGURE 3.**
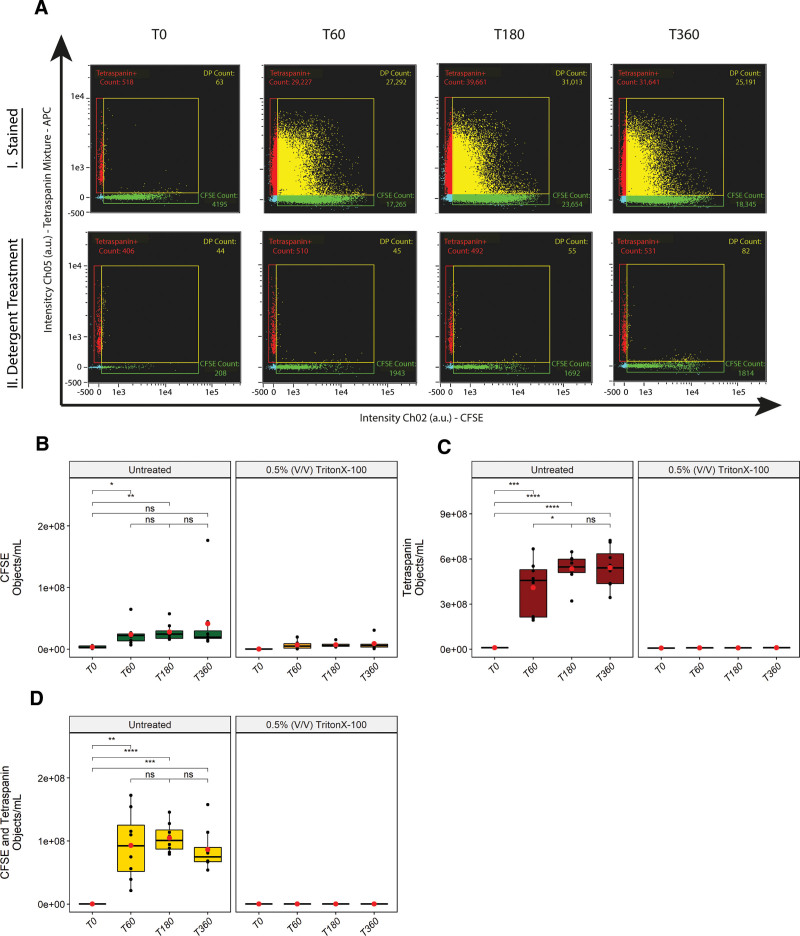
EV release by discarded kidneys during NMP. A, Representative scatter plots of a stained sample (top row) and the corresponding sample after detergent treatment (bottom row) for each time point measured (T0/T60/T180/T360, min). Concentrations of single-positive CFSE (B), single-positive tetraspanin (C), and double-positive CFSE and tetraspanin fluorescent objects/mL (D) before and after detergent treatment per time point measured. Red dots/black lines representing the means/median values of each time point, respectively. Statistical analysis (unpaired *t*-test, two sided [*P < 0.05, **P < 0.01, ***P < 0.001, ****P < 0.0001]) showed significant release of EV for all time points with respect to pre-NMP samples (C and D). Only the tetraspanin single-positive population (C) showed a significant increase from 60 to 180 min. No significant differences in release were observed between the other time points/populations. CFSE, carboxyfluorescein succinimidyl ester; EV, extracellular vesicle; NMP, normothermic machine perfusion.

For the stained samples, we observed CFSE and tetraspanin single-positive—but very few double-positive (<70 events)—fluorescent background events in perfusate samples drawn before exposure to the kidney (T0). Samples collected after 60/180/360 min of NMP showed increases in fluorescent events across all 3 populations (CFSE single-positive, tetraspanin single-positive, and CFSE and tetraspanin double-positive). Detergent treatment was applied on each sample after initial acquisition to discriminate between vesicular and nonvesicular events (Figure 3A).

First, concentrations of fluorescent objects before and after detergent treatment were compared for each time point. For CFSE single-positive objects we observed a ~69%/72%/76% reduction in concentration after detergent treatment at T60/T180/T360, respectively (Figure 3B). This implies that a large fraction (~31%/28%/24%) of these objects represent nonvesicular (background) objects as they had not been dissolved by the detergent treatment. Consequently, the CFSE single-positive population was excluded from further analysis. Detergent treatment reduced tetraspanin single-positives (Figure 3C) and CFSE and tetraspanin double-positives (Figure 3D) with 97.7% ± 0.004% and 99.8% ± 0.0002%, respectively (normalized mean ± SD, average reduction over all time points). Background levels (concentrations obtained after detergent treatment) of the Tetraspanin single-positive population resided around ~E^6^ objects/mL whereas the level of CFSE and Tetraspanin double-positives were observed to be <E^5^ objects/mL. These background levels were comparable to the baseline perfusate (T0) samples before detergent treatment.

Second, for tetraspanin single-positive and CFSE and tetraspanin double-positive populations we observed ~43/56/57 and ~507/572/471—fold increases after 60/180/360 min of NMP compared to T0, respectively (comparison of means). Comparing the mean concentration of tetraspanin single-positive events to the mean concentration of tetraspanin and CFSE double-positive events revealed ~4/5/6—fold differences at 60/180/360 min of NMP respectively—indicating that less CFSE-positive EVs were detected as NMP progressed.

Taken together, these findings indicate that (1) kidneys release EVs during NMP and (2) different subpopulations (based on tetraspanin expression in combination with the absence/presence of CFSE) can be identified using IFCM.

### EVs Released During NMP Express Predominantly CD81

**FIGURE 4.**
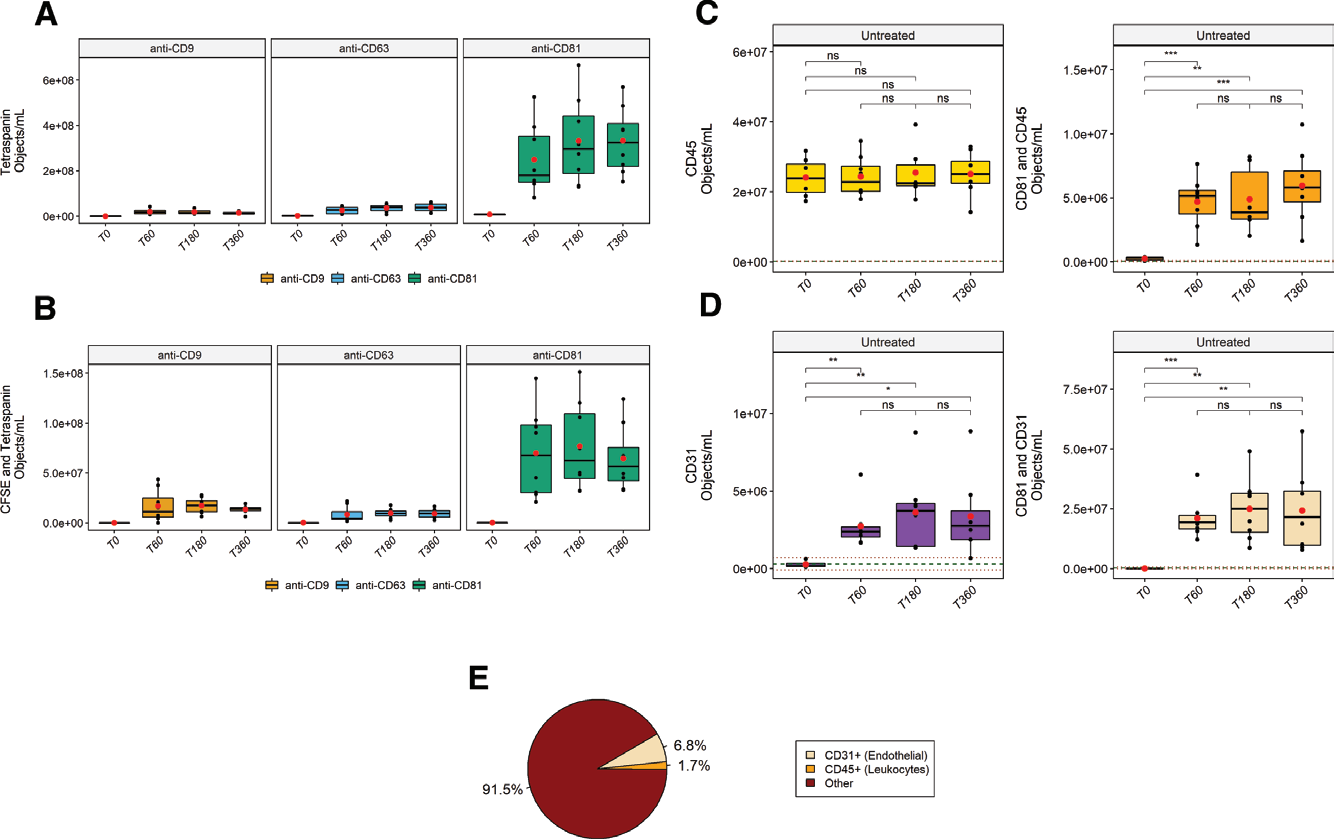
Phenotyping and determination of the concentration of EVs released during NMP. NMP samples were stained with CFDA-SE in combination with anti-CD9, anti-CD63, or anti-CD81, acquired using IFCM and analyzed using the previously described gating strategies. A, Tetraspanin profile of tetraspanin single-positive objects. B, Tetraspanin profile of double-positive objects. Additionally, perfusates were stained with anti-CD81 (predominantly expressed on EVs released during NMP) in combination with (C) anti-CD45 (as pan-leukocyte marker) or (D) CD31 (as prominent endothelial marker). Significantly increased levels of EVs were detected compared to T0 for all identified subpopulations (*P < 0.05, **P < 0.01, ***P < 0.001, and ****P < 0.0001), with the exception of CD45 single-positive EVs. Red dots/black lines represent the means/median values of each time point, respectively. Dashed lines: mean (green) ± SD (dark red) of respective isotype controls (shown for C and D). E, Assessment of cellular origin of detected CD81+ EVs. For all time points and samples, the fraction of double-positive events compared to total detected CD81+ events (both single-positive and double-positive events) was calculated and averaged to determine the relative abundance of double-positive EVs for either population, thus allowing deduction of cellular origin. CFDA-SE, carboxyfluorescein diacetate succinimidyl ester; CFSE, carboxyfluorescein succinimidyl ester; EV, extracellular vesicle; IFCM, imaging flow cytometry; NMP, normothermic machine perfusion.

Following the identification of EVs based on tetraspanin expression, we examined the tetraspanin distribution on the released EVs by staining the NMP samples with CFDA-SE and one of the individual components of the antitetraspanin antibody mixture at a concentration equal to that used within the mixture. We observed that CD81+ EVs represented ~86% and ~74% of single and double-positive fluorescent events, respectively, across the time points analyzed (normalized average of time points 60, 180, and 360 min, Figure 4A,B). CD9+ and CD63+ EVs were found to represent ~5% and ~9% of the detected single-positive fluorescent events and 16% and 9% of the double-positive fluorescent events, respectively.

These findings show that tetraspanin CD81 is predominantly expressed on EVs released during NMP. Additionally, CFSE fluorescence was detected in conjunction with all tetraspanins studied, indicating that esterase activity is not exclusively linked to any of these tetraspanins.

### Leukocyte and Endothelial-derived EVs Are Released During NMP

Surface proteins on EVs reflect the biological origin of their parental cells and next we determined the expression of either CD45 (as a pan-leukocyte marker) or CD31 (as a prominent endothelial marker) on the CD81+ EVs. We performed double staining of the perfusates with anti-CD45 or anti-CD31 in combination with anti-CD81 and analyzed each fluorescent population. For the CD45 single-positive events, ~52% of the events were still present after detergent treatment (data not shown) and no significant increases were observed during NMP when compared to baseline perfusates (T0 – 2.7E^7^ ± 5.4E^6^ objects/mL) despite high specificity of the mAbs as indicated by isotype controls (dashed lines – 1.7E^5^ ± 9.8E^4^ objects/mL, Figure 4C). Thus, CD45 single-positive EVs could not be discriminated from baseline perfusate signals. Analysis of CD81 and CD45 double-positive events yielded ~97.5% reduction after detergent treatment, a significant 18/19/23-fold difference in objects/mL at 60/180/360 min of NMP compared to T0 (2.6E^6^ ± 1.4E^5^ objects/mL), and high specificity as indicated by isotype controls (4.3E^4^ ± 4.1E^4^ objects/mL, Figure 4C).

Analysis of CD31 single-positive events showed ~91% reduction after detergent treatment, 11/ 14/ 13 – fold difference in objects/mL at each time point of sample drawing compared to T0 (2.6E^5^ ± 1.8E^4^ objects/mL), and an isotype background of 6.39E^5^ ± 4.67E^5^ objects/mL. For CD81 and CD31 double-positive events, we observed >99% reduction of fluorescent events after detergent treatment, ~950/ 1130/ 1100 – fold difference in objects/mL at each time point of sample drawing compared to T0 (2.2E^4^ ± 1.9E^4^ objects/mL), and an isotype background of 3.28E^4^ ± 1.53E^4^ objects/mL (Figure 4D).

We then analyzed the relative abundance of both double-positive EV populations with respect to the total CD81+ EVs detected. We observed that 6.8% of CD81+ EVs expressed the endothelial cell marker CD31, and only 1.7% of CD81+ EVs was found to express the common leukocyte antigen CD45 (Figure 4E). The far majority of CD81+ EVs detected (91.5%) were found to not express either of the measured markers.

In summary, these data show that leukocyte and endothelial-derived EVs are released during NMP. Surprisingly, the majority of CD81+ EVs did not bear either of the studied markers.

### Concentrations of Released EV Subsets Are Correlated With Donor Demographics and NMP Viability Characteristics

To determine whether the identified EV subsets can be used as indicators of kidney quality before transplantation, we performed a correlation analysis between the concentrations of EVs released by each individual kidney, and—in the absence of posttransplantation kidney function—donor kidney characteristics (specified in Table 1) and NMP viability characteristics (as a surrogate for kidney quality specified in Table 2).

**TABLE 2.** NMP viability characteristics as measured at 60/180/360 min of NMP

Kidney ID	K1*^a^*	K2*^a^*	K3	K4	K5	K6	K7*^b^*	K8*^b^*
Renal blood flow (mL/min/100 g)								
T60	16.54	84.66	83.02	12.14	30.54	23.38	16.58	25.82
T180	31.20	84.66	93.37	25.63	36.24	35.71	24.39	31.92
T360	45.11	91.48	151.19	30.44	47.99	66.23	58.54	92.96
Intrarenal vascular resistance (mm Hg/mL/min)								
T60	1.7	0.25	0.24	1.19	0.82	1.04	1.1	1.36
T180	0.9	0.25	0.21	0.56	0.69	0.68	0.75	1.10
T360	0.63	0.23	0.13	0.47	0.52	0.37	0.31	0.38
Total urine production (mL) – accumulated								
T60	0	71.5	63	0	0	0	0	0
T180	0	100.5	120	0	3	12	0	0
T360	0	116	303	0	3	48	0	0
Transplantability assessment post NMP	No	No	Yes	No	Yes	No	No	No

After 6 h of NMP, each ECD kidney was judged by an independent transplant surgeon and nephorologist whether or not the organ was deemed suitable for transplantation (transplantability assessment).

*^a-b^*Represent paired kidney grafts from same donor.

ECD, expanded-criteria donor; ECD, expanded-criteria donor; NMP, normothermic machine perfusion.

**FIGURE 5.**
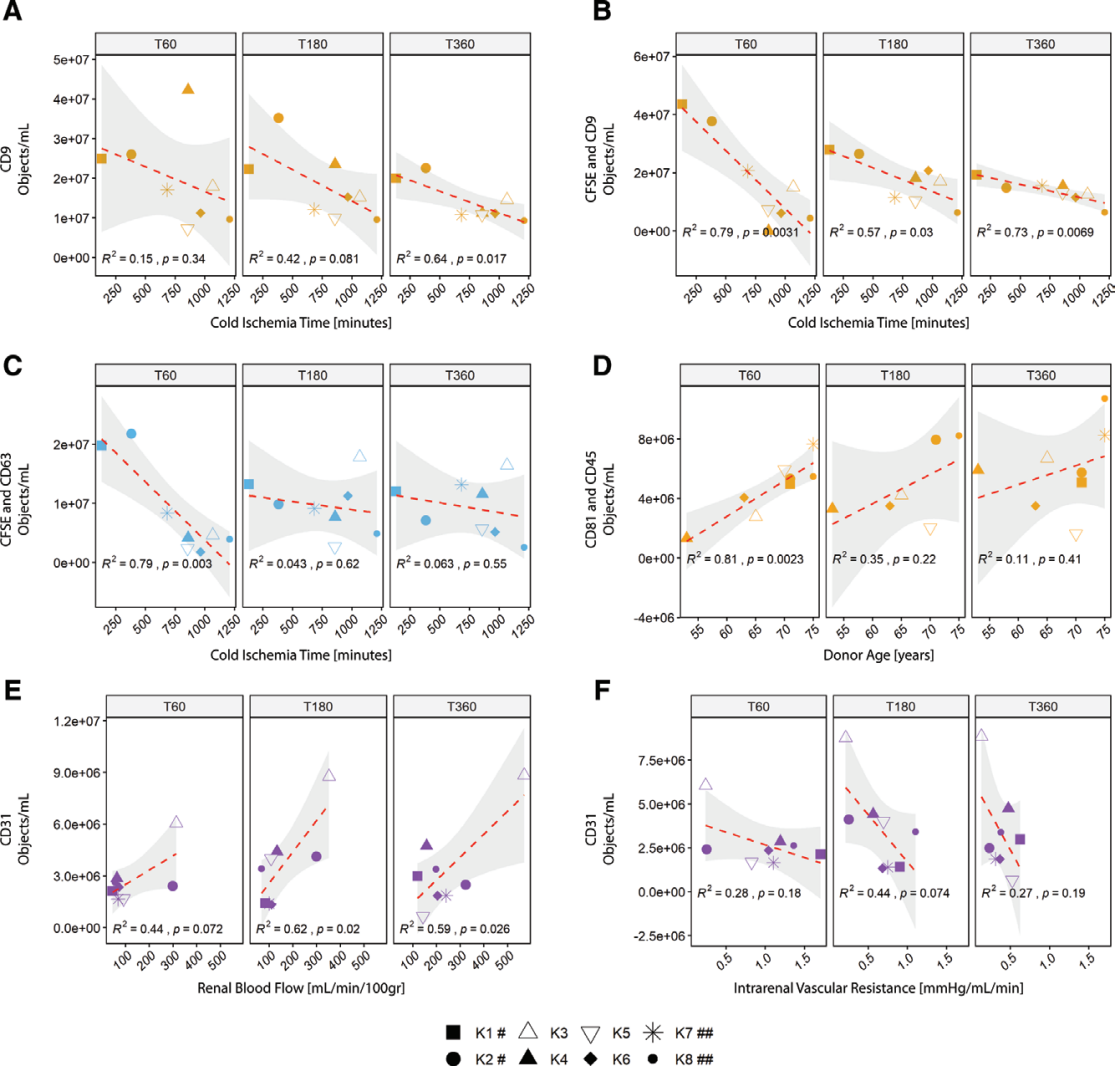
Correlation analysis of concentrations of released EV subsets with donor kidney and NMP viability characteristics. Overview of the EV subsets for which statistically significant correlations were obtained. Analysis of (A) correlation between CD9 single-positive, (B) CFSE and CD9 double-positive, and (C) CFSE and CD63 double-positive EVs with CIT. D, Analysis of correlation between CD81 and CD45 double-positive EVs with donor age. Most notably, correlation analysis of CD31 single-positive EVs with (E) renal flow and (F) intrarenal vascular resistance showed inverse correlations. EV concentrations as excreted by each ECD kidney are represented by shape, with open triangles representing K3 and K5 (which were deemed transplantable post-NMP – Table 2). *^a^* and *^b^* represent paired kidney grafts from same donor. CFSE, carboxyfluorescein succinimidyl ester; CIT, cold ischemia time; ECD, Expanded-criteria donor; EV, extracellular vesicle; NMP, normothermic machine perfusion.

Analysis of anti-CD9 single-positive EVs and CIT revealed a significant correlation after 360 min of NMP (R^2^ = 0.64, *P* = 0.017, Figure 5A), whereas no significant correlations with CIT were obtained for any of the other single-positive EV subsets. For CFSE and anti-CD9 double-positive EVs, significant correlations were observed for all time points analyzed (*P* < 0.05, Figure 5B). Additionally, CFSE and anti-CD63 double-positive EV were found to be significantly correlated after 60 min (R^2^ = 0.79, *P* = 0.003)—but not after 180 and 360 min—of NMP (Figure 5C). Analysis of CD81 and CD45 double-positive EVs revealed a positive correlation with donor age after the first 60 min of NMP only (R^2^ = 0.81 and *P* = 0.0023, Figure 5D). Anti-CD31 single-positive EVs were found to be the only EV subset that showed significant correlations with an NMP viability characteristic. For all time points analyzed, a positive correlation between concentrations of CD31+ EVs and renal blood flow was observed (Figure 5E), whereas negative correlations were found with intrarenal vascular resistance (Figure 5F). Although we did observe trends between some of the other donor kidney characteristics or NMP viability characteristics (eg, kidney weight or impact of initial cold preservation—static cold storage versus HMP) and EV subset concentrations, none were found to be statistically significant.

Additionally, we did not observe any divergences in EV subset concentrations released by matched kidney grafts retrieved from the same donor (K1 and K2, K7 and K8), or kidneys that were deemed transplantable after 6 h of NMP (K3 and K5), when analyzing each kidney individually (as represented by the different shapes in Figure 5).

Altogether, these findings indicate that the release of the identified EV subsets are differentially correlated to donor kidney characteristics and NMP viability characteristics.

## DISCUSSION

A major benefit of machine perfusion is that it allows for the assessment of kidney quality before transplantation through the analysis of biomarkers (such as EVs) in the perfusion fluid.^2,5,7^ Especially during NMP, where cellular metabolism becomes activated, the monitoring of EVs may be a promising tool to infer kidney quality before transplantation.^26^ Additionally, the characterization of EVs released during NMP may shed light on the origin and composition of EVs released into circulation of transplant recipients and increase our understanding of (distal) immune responses.^27-29^ However, although EVs are subject to intensive biomarker studies in various fields,^13,30,31^ little is currently known regarding EV release by kidney grafts during NMP and its association with kidney status.

In this observational study, we examined and characterized the release of EVs by discarded human ECD kidneys during NMP. In line with our previous findings,^22^ we found that the majority of released nanoparticles were <400 nm in size irrespective of the time of sampling and that total particle concentrations increased as NMP progressed. Using IFCM we found that EV concentrations significantly increased during the first 60 min of NMP and that concentrations remained relatively stable during the remainder of the NMP procedure (up to 6 h). We reason that the observed stabilization of EV concentrations is due to the establishment of an equilibrium between EV biogenesis and breakdown during NMP and/or uptake by (endothelial) cells of the kidney. These findings may contribute to the debate regarding optimal NMP perfusion times: since EV release is considered an active process,^32^ release dynamics during NMP may be dependent on the metabolic status of the kidney graft.

Examination of the tetraspanin profile on the released EVs revealed that the majority of detected EVs expressed tetraspanin CD81. Recently, CD81 has been shown to serve as a regulator of B cell signaling through complex formation with CD19 at the plasma membrane. Upon B cell activation CD19 dissociates from CD81 whereas in naïve B cells CD81 (epitope 5A6) is complexed by CD19.^33^ Given that the majority of detected EVs released during 6 h of NMP expressed the CD81 epitope 5A6, the fusion of these CD81+ EVs with recipient B cells may have a dampening effect on B cell signaling as the addition of extra CD81 onto the B cell membrane may affect CD81-CD19 dissociation kinetics. Additionally, it has been shown that allograft-derived EVs bearing intact donor major histocompatibility complex molecules (CD63+ and CD9+CD81+ EV subsets) are able to cross-decorate and activate alloreactive recipient B cells in a mouse skin-transplant model.^29^ In a human setting, this cross-decoration might be facilitated by CD81+ EVs.

When determining the origin of detected CD81+ EVs, we found only marginal colocalization of CD81 with endothelial and hematopoietic markers CD31 and CD45. However, part of the CD81+CD31+EV may represent CD45+EVs derived from monocytic origin. Additionally, the low percentages of colocalization may be influenced by the usage of mAbs targeting extravesicular epitopes^20^ (thus ignoring the presence of markers on the luminal side of EV). Moreover, the released particles were determined to be <400 nm (as shown by NTA, and selected in the IFCM analysis) and therefore were assumed to consist largely of exosomes. Exosomes form through inward budding of the membrane of early endosomes (forming multivesicular bodies in the process), which eventually fuse with the cell plasma membrane, releasing its content into the extracellular space.^34^ Consequentially, it is hypothesized that not all exosomes necessarily bear parental cell surface markers—which may explain why >90% of CD81+ EVs were found to not be colocalized with either anti-CD31 or anti-CD45.

Correlation analysis of CFSE+EVs and CIT revealed negative correlations for EVs bearing tetraspanins CD9 or CD63 (during the first 60 min of NMP)—but not CD81. Since CFDA-SE needs intravesicular esterases to acquire its fluorescent properties (CFSE+), these negative correlations may be explained by (1) the negative impact of CIT on cellular (and thus vesicular) enzyme (esterase) activity or (2) reduced release of EVs containing intravesicular esterases as a consequence of CIT. Diminished correlations of CFSE+ and CD9+/CD63+ double-positive EVs with CIT were found after 180 and 360 min of NMP—which might be explained by the restoration of cellular metabolism during the course of NMP.^5,26^

As the perfusion pressure during NMP is fixed, the perfused kidneys autoregulate their blood flow according to intrarenal vascular resistance. The inverse correlation between low renal blood flow and high intrarenal vascular resistance during NMP has been described in the literature and has been associated with increased vascular injury or interstitial edema.^35^ We demonstrate that the release of CD31+ single-positive EVs (likely to be of endothelial origin) is positively correlated with renal blood flow, and consequently inversely correlated with intrarenal vascular resistance. As it is well-known that the endothelial cell layer of microvessels is a key modulator of vasodilation through the synthesis and release of vasoactive substances,^36^ CD31 single-positive EVs might be indicative for kidney quality before transplantation. Recently, to aid clinicians in determining kidney quality during NMP, a scoring system has been developed based on the macroscopic appearance and thresholds of renal blood flow and urine output.^35^ The presence of different EV subsets (such as CD31+ EVs) might be added—after extensive validation in transplanted cohorts—to this scoring system. Potential identification of other EV subsets and their correlation with kidney function post KTx may provide insight into (1) kidney quality and (2) the specific compartment(s) of the kidney which are injured/functioning sub-optimally before transplantation. However, in the current work, correlation analysis of the other identified EV subsets with donor kidney characteristics or NMP viability markers did not result in any statistically significant correlations; either when analyzed as a group or when examining EV release for each kidney individually. Although K3 and K5 were determined to be of sufficient quality to be transplantable after 6 h of NMP, we did not observe any trends that differentiated these kidneys from the other ECD kidneys during NMP for any of the markers examined in this study.

It must be noted that no direct inferences could be made between kidney quality before transplantation (or transplant outcome) and the released EV subsets as none of the kidneys studied were actually transplanted. Additionally, as these kidneys represent ECD kidneys the reported concentrations and observed correlations might be different for non-ECD kidneys. Another limitation of this study is the small sample size: the heterogeneity among the ECD kidneys with respect to for example, cause of donor death and the type of organ storage might (further) impact the amount and/or subsets of EVs released. However, the observed correlations of different EV subsets with CIT and donor age during the first 60 min of NMP do indicate that EV release is related to well-established indicators of kidney quality: both CIT and donor age are known to be detrimental to kidney quality/transplant outcome.^4,37^

In conclusion, our findings demonstrate that discarded human ECD kidney grafts release different EV subsets during NMP and that their release is correlated with well-established indicators of kidney quality such as CIT, donor kidney age, renal blood flow and intrarenal vascular resistance. The identification, quantification and phenotyping of kidney-derived EVs released during NMP may represent a starting point to study the role of EVs as potential biomarkers for kidney graft quality before transplantation. How and if the identified (and other) EVs subsets are correlated with kidney function posttransplantation will be the focus of future research.

## ACKNOWLEDGMENTS

The authors would like to thank the members of the PROPER Consortium (PROlonged ex vivo normothermic machine PERfusion for kidney regeneration) for setting up the study, provision of the normothermic machine perfusion samples and input in research design: Dr Ian Alwayn, Dr Rutger Ploeg, Dr Dorottya de Vries, Dr Volkert Huurman, Jason Doppenberg, and Asel Arykbaeva from the Leiden University Medical Center; Dr Henri Leuvenink, Dr Robert Pol, Dr Cyril Moers, Tim Hamelink, Veerle Lantinga, and Leonie van Leeuwen from University Medical Center Groningen; and Dr Robert Minnee from the Erasmus Medical Center, Rotterdam, The Netherlands. Additionally, the authors would like to thank Manou van Alphen for her assistance with NMP sample measurements with IFCM.

## Supplementary Material


