# Supplementary material for: Extracellular Vesicles Released During Normothermic Machine Perfusion Are Associated With Human Donor Kidney Characteristics
Source: Transplantation. 2022 Nov 22;106(12):2360–9. doi: 10.1097/TP.0000000000004215 (PMC9698093; doi:10.1097/TP.0000000000004215)
Supplement: Supplementary file 1 [file tp-106-2360-s001.pdf]

SUPPLEMENTAL DIGITAL CONTENT

Sample Labeling S1

EVs released by ECD kidneys during NMP were phenotoyped and quantitated with Imaging Flow Cytometry. Samples were labelled with an anti-tetraspanin antibody mixture (consisting of anti-CD9/anti-CD63/anti-CD81), anti-CD31, anti-CD45, or CFDA-SE. All mAbs used - (CD9–APC, clone HI9a (6 µg/mL, Biolegend), CD63–APC, clone H5C6 (200 µg /mL, Biolegend), CD81–APC, clone 5A6 (200 µg /mL, Biolegend), CD31–BV421, clone WM-59 (50 µg/mL, BioLegend), CD45-PE, clone HI30 (12.5 µg/mL, BD Biosciences), IgG1,k-BV421, clone MOPC-21 (100 µg/mL, BioLegend); IgG1,k-APC, clone MOPC-21 (200 µg/mL, BioLegend); and IgG1,k-PE, clone MOPC-21 (12.5 µg/mL, BD Biosciences) - were centrifuged at 16,000 x *g* (to remove potential mAb clumps).Tetraspanin mAbs were diluted 30-fold in fPBS before staining (Final concentrations: CD9: 200 ng/mL, CD63: 6.6 µg/mL, CD81: 6.6 µg/mL); CD31-BV421 was diluted 1000-fold (Final concentration: 50 ng/mL); CD45-PE was used undiluted (Final concentration: 12.5 µg/mL).

A 50 µM Carboxyfluorescein diacetate succinimidyl ester stock solution (Vybrant^{TM} CFDA-SE Cell Tracer Kit, Invitrogen) was prepared according to the manufacturers' instructions and, similar to the mAbs, centrifuged for 10 minutes at 16,000 x *g*. Staining was performed by adding 30 µL of perfusate followed by addition of the diluted mAbs into a pre-determined volume of fPBS (V_{tot} = fPBS + sample + mAbs = 130 µL) followed by O/N incubation at 4 ^{0}C, with 2.5 ng anti-CD9, 83 ng anti-CD63, 83 ng antiCD81, 0.25 ng anti-CD31, and 156 ng anti-CD45 per test. Staining with CFDA-SE was performed by

adding 100 µL of the stock solution to the samples followed by 30 minutes of incubation at room temperature in the dark. All samples were brought to a total volume of 380 µL using fPBS before IFCM measurements.

Acquisition S1

Perfusate samples were interrogated with IFCM (ImageStreamX MkII, Luminex) to phenotype and quantitate Extracellular Vesicles. Lasers were turned on as applicable per fluorophore and set to their maximum power (488 nm : 200 mW, 642 nm :150 mW) with the exception of the 785 nm SSC laser (1.25 mW). Data was acquired over a time period of 180 seconds – to standardize among samples – using the 60x objective with fluidics set to ‘low speed / high sensitivity’. This resulted in a flow speed of 43.59 ± 0.07 mm/sec (mean ± standard deviation). Core size was set at 6 µm, autofocus was activated and the ‘Remove Speedbead’ was option unchecked.

BV421 fluorescence signals were collected in channel 1 (435–505-nm filter), CFSE signals were collected in channel 2 (505–560 nm filter), PE fluorescence signals were collected in channel 3 (560–595-nm filter), APC signals in channel 5 (642–745 nm filter), and SSC signals in channel 6 (745–785 nm filter). Particle enumeration was achieved through the advanced fluidic control of the IS^x coupled with continuously running speed beads, resulting in the “objects/mL” feature within the IS^x Data Exploration and Analysis Software (IDEAS®).

Figure S1

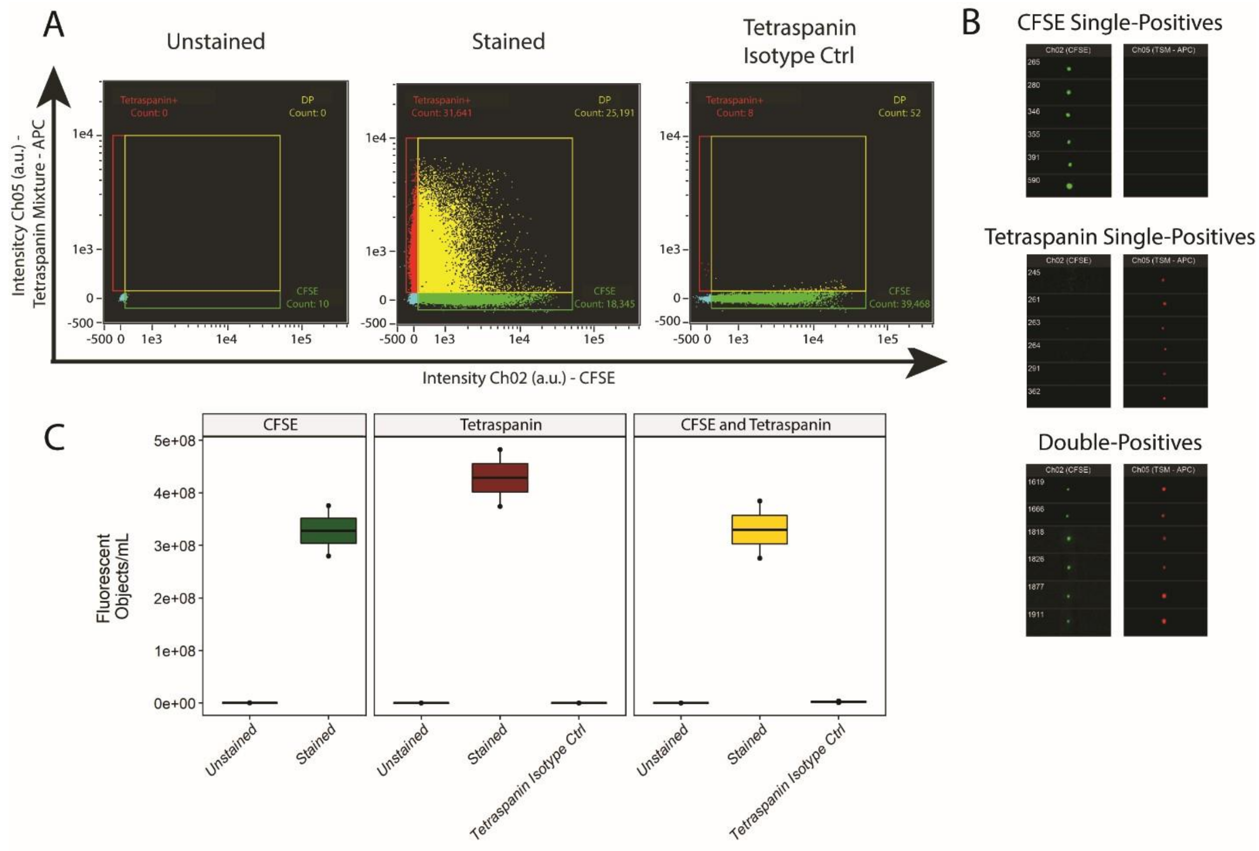

Figure S1 – Identification and validation of single EV measurements using Imaging Flow Cytometry (IFCM). A) From left to right: fluorescence intensity scatterplots representative for unstained, stained (CFDA-SE & anti-tetraspanin antibody mixture (anti-CD9/anti-CD63/anti-CD81)), and isotype control end-point NMP perfusate samples (T = 360 minutes). Fluorescent populations were established on the basis of unstained and single-stained end-point NMP samples (data not shown). B) Visual examination of events representative for each fluorescent population demonstrated the selection and subsequent analysis of particles showing single spot fluorescence (no coincidence events), indicating the selection and analysis of single EVs. C) Quantification of fluorescent events in each gate for unstained, stained and isotype control (N=2),

showing the specificity of our staining protocol. In unstained samples, no fluorescent events were observed indicating that no auto-fluorescent events were detected. In stained samples, we observed concentrations of fluorescent events ($>E^{8}$ objects/mL) well above unstained and isotype levels ($<E^{5}$ Objects/mL), indicating that the fluorescent events acquired in samples stained with the anti-tetraspanin antibody mixture represent specific fluorescent positive events.
